# Circulating microRNAs as a potential biomarker for osteoporosis in patients with type 2 diabetes mellitus: a retrospective clinical study

**DOI:** 10.3389/fendo.2025.1534725

**Published:** 2025-08-04

**Authors:** Yuqi Li, Lu Gan, Dan Zhao, Hong Lei, Liping Sha

**Affiliations:** Department of Endocrinology, Cardio-Cerebrovascular Disease Hospital, General Hospital of Ningxia Medical University, Yinchuan, Ningxia, China

**Keywords:** type 2 diabetes mellitus, osteoporosis, miRNA, early diagnosis, diabetic osteoporosis (DOP)

## Abstract

**Objective:**

By analyzing the expression levels of circulating microRNAs (miRNAs) in patients with type 2 diabetes mellitus (T2DM) and its correlation with diabetic osteoporosis (DOP), this study aims to identify potential biomarkers for the early prediction and screening of DOP.

**Methods:**

A total of 120 patients with T2DM who received treatment in the endocrinology outpatient/inpatient department between January 2023 and June 2024, along with 90 healthy volunteers, were enrolled in this study. Based on the bone mineral density (BMD), the 120 T2DM patients were divided into three groups: normal group (54 cases), osteopenia group (38 cases), and osteoporosis group (28 cases). The differences in clinical data, laboratory test indicators and miRNA expression differences among the three groups were statistically analyzed, and the high-risk factors for DOP in T2DM patients were analyzed.

**Results:**

Compared to healthy volunteers, patients with T2DM demonstrated significantly decreased levels of P1NP and miR-219a-5p, alongside elevated levels of β-CTX, miR-188-3p, and miR-19a/b. Additionally, miR-335-5p levels were notably reduced in T2DM patients. Among these markers, significant differences were observed in the expression levels of P1NP, β-CTX, and miRNA in T2DM patients. Further analysis revealed distinct expression patterns of miR-188-3p, miR-335-5p, and miR-19a/b across the three T2DM subgroups (osteoporosis, osteopenia, and normal bone density groups). Specifically, miR-188-3p levels were 10.34 ± 1.26 in the osteoporosis group, 8.35 ± 1.33 in the osteopenia group, and 6.55 ± 1.18 in the normal group. Similarly, miR-335-5p levels were 0.44 ± 0.14, 0.67 ± 0.16, and 0.88 ± 0.15, respectively, while miR-19a/b levels were 4.04 ± 1.41, 3.19 ± 1.21, and 2.47 ± 1.24, respectively (P < 0.001 for all comparisons). These miRNAs also exhibited significant correlations with BMD at the hip and lumbar spine (P < 0.001 or P = 0.001), highlighting their potential role in bone metabolism and osteoporosis risk in T2DM patients.

**Conclusions:**

The results suggest that the circulating levels of miR-188-3p, miR-335-5p, and miR-19a/b are significantly associated with the occurrence of DOP in T2DM patients. These miRNAs show potential as biomarkers for the early diagnosis of DOP.

## Introduction

With the improvement of living standards and changes in lifestyle, the prevalence of diabetes mellitus (DM) has increased rapidly, and the total number of diabetes patients in China ranks second in the world (1). Among them, type 2 diabetes mellitus (T2DM) accounts for 93.7% of the diabetic population (2). As understanding and research on diabetes and its complications have deepened, the changes in bone metabolism caused by diabetes have gradually received attention. Diabetic osteoporosis (DOP) is a type of disease caused by changes in bone metabolism caused by diabetes, which leads to a decrease in bone mass and destruction of bone structure, and ultimately increases risk of fractures. DOP is considered a type of secondary osteoporosis (3, 4). The number of patients in China continues to increase, and more and more people are suffering from reduced bone mass. If a fracture occurs, it will place a heavy burden on the patient’s family and cause physical and psychological trauma (5, 6).

Currently, the clinical diagnosis and efficacy evaluation of DOP are mostly based on fragility fractures and/or bone mineral density (BMD) measured by dual-energy X-ray absorptiometry (DXA) (7). It may be time-consuming to diagnose DOP in the absence of a fragility fracture, and the occurrence of the first fragility fracture also increases the risk of subsequent fractures (8). Early detection and timely clinical intervention in patients with osteoporosis are crucial, as they can substantially mitigate the risks of fractures, thereby preventing physical and psychological trauma to patients. Additionally, such measures can alleviate the economic burden on healthcare systems and society at large. Consequently, the early identification and targeted prevention and management of osteoporosis hold significant clinical and public health importance.

MicroRNAs (miRNAs) are a class of short noncoding single-stranded molecules, typically 18–24 nucleotides in length, that play a key role in gene translation and expression by binding to the 3’untranslated region (3’UTR) of target messenger RNAs (mRNAs) (9). A large number of studies have shown that osteoporosis is associated with abnormal expression of miRNA in bone tissue and blood circulation, and miRNA is involved in the pathogenesis of osteoporosis (10). miRNA may be a potential biomarker for predicting the prognosis of high-risk osteoporotic fractures (11). However, its role in DOP is still unknown. Therefore, this study aims to explore the correlation between circulating miRNA levels and the occurrence of DOP to provide theoretical guidance and basis for early prediction and screening.

## Materials and methods

### Participants

This study retrospectively collected data from 120 patients with T2DM who were treated by the endocrinology department of Cardio-Cerebrovascular Disease Hospital, General Hospital of Ningxia Medical University between January 2023 to June 2024, along with 90 healthy volunteers who were randomly included in the study. The diagnostic criteria for T2DM were as follows: (1) fasting blood glucose levels ≥ 7mmol/L, measured at least twice; (2) typical symptoms such as polydipsia, polyuria, polyphagia, and weight loss, with random blood glucose levels ≥ 11.1 mmol/L; (3) 2h blood glucose levels ≥ 11.1 mmol/L during an oral glucose tolerance test (OGTT) (12). Based on the diagnostic criteria for osteoporosis, the BMD of the patients was measured using dual-energy X-ray absorptiometry (DEXA, equipment Model: Hologic Discovery Wi) to calculate the T-scores. DXA machine calibration follows strict manufacturer’s calibration guidelines and DXA equipment is regularly calibrated and maintained. All operators involved in dual-energy X-ray absorptiometry (DXA) measurements undergo rigorous training and adhere to standardized operating procedures to ensure consistency, accuracy, and reliability in data acquisition and interpretation. The 120 patients with T2DM included in the study were divided into three groups: osteoporosis group (T-score ≤ -2.5 standard deviations), osteopenia group (T-score between -2.5 and -1.0 standard deviations), and normal group (T-score ≥ -1.0 standard deviations) (13). The T-score diagnostic criteria used in this study were based on the 2020 American College of Clinical Endocrinologists/American College of Endocrinology (AACE/ACE) Guidelines for the Management of Postmenopausal Osteoporosis. The study was approved by the Ethics Committee of General Hospital of Ningxia Medical University (KYLL-2022-0693).

### Collection of clinical data and laboratory test indicators

General clinical data were collected from the study subjects, including age, gender, duration of diabetes, height, weight, body mass index (BMI), and clinical history. Fasting venous blood samples were collected from patients to test for laboratory indicators, including fasting blood glucose (FBG), glycosylated hemoglobin (HbA1c), triglyceride (TG), low-density lipoprotein (LDL), high-density lipoprotein (HDL), vitamin D3, parathyroid hormone (PTH), bone mineral density (BMD), procollagen type I N-terminal propeptide (P1NP), and serum β-Cross Linked C-telopeptide of type I collagen (β-CTX). All tests were performed by the laboratory of our hospital and the relevant results were issued.

### Quantitative detection of miRNA (miR-219a-5p, miR-188-3p, miR-335-5p and miR-19a/b)

Fasting venous blood samples (5 mL) was collected from patients or healthy volunteers within 12 hours of admission, and the serum was separated using a centrifuge (WIGGENS, Germany, model: UNICEN21) and stored at -80°C for later use. The miRNA in serum was extracted using the EasyPure^®^ miRNA Kit (Beijing TransGen Biotech, ER601-01-V2), and the concentration and purity were determined by a spectrophotometer (Dongguan Pubiao Technology Co., Ltd.). The miRNA was reverse transcribed into cDNA by the tailing method using a reverse transcription kit (Takara, 638315). U6 was used as the internal reference gene of miRNA, and the primer sequences are shown in **Table 1**. The reaction system was prepared according to the instructions of the quantitative kit (Takara), and the PCR amplification conditions were set as follows: 95°C for 30 seconds, 95°C for 15 seconds, 60°C for 30 seconds, 72°C for 30 seconds, for 40 cycles. The relative expression levels of miRNA were analyzed using the 2-ΔΔCt method.

**Table 1:** Comparison of clinical characteristics among the three groups.

Characteristic	Osteoporosis (N=28)	Osteopenia (N=38)	Normal (N=54)	*P* value
Gender (Male/Female)	16/12	22/16	36/18	0.674
Age (years)	62.59 ± 6.50	65.58 ± 6.77	65.87 ± 7.46	0.118
Duration (years)	9.71 ± 6.02	12.27 ± 4.61	10.18 ± 6.58	0.146
BMI (kg/m^2^)	23.70 ± 3.03	22.59 ± 2.94	23.59 ± 2.34	0.675
Medical history, n (%)				
Myocardial infarction	6 (21.42%)	8 (21.05%)	14 (25.92%)	0.831
Hypertension	8 (28.57%)	15 (39.47%)	26 (48.14%)	0.528
Hyperlipidemia	4 (14.28%)	7 (18.42%)	13 (24.07%)	0.551
Cardiovascular disease	12 (42.85%)	16 (42.10%)	29 (53.70%)	0.468
Metabolic syndrome	9 (32.14%)	13 (34.21%)	16 (29.62%)	0.896
FBG (mmol/L)	6.07 ± 1.75	6.69 ± 1.54	6.22 ± 1.53	0.234
HbA1c (%)	10.11 ± 1.96	9.60 ± 1.36	9.50 ± 1.36	0.212
TG (mmol/L)	1.64 ± 0.61	1.52 ± 0.73	1.52 ± 0.70	0.752
HDL (mmol/L)	1.05 ± 0.37	1.10 ± 0.26	1.14 ± 0.32	0.515
LDL (mmol/L)	2.42 ± 0.54	2.04 ± 0.60	2.31 ± 0.59	0.053
VitD3 (nmol/L)	8.18 ± 1.99	8.71 ± 1.86	9.23 ± 2.53	0.120
PHT (pg/mL)	38.27 ± 11.84	42.48 ± 8.81	37.93 ± 10.02	0.087
T-score of BMD				
Lumbar spine (%)	-2.94 ± 0.85	-1.03 ± 0.73	0.91 ± 1.10	<0.001
Right hip (%)	-2.29 ± 1.10	-1.19 ± 0.96	0.08 ± 0.87	<0.001
Left hip (%)	-2.32 ± 1.06	-1.16 ± 0.88	0.30 ± 0.83	<0.001
P1NP (ng/mL)	38.98 ± 5.53	39.36 ± 5.17	41.17 ± 6.50	0.189
β-CTX (pg/mL)	429.70 ± 10.56	391.20 ± 50.13	357.91 ± 38.99	<0.001
miR-219a-5p	0.68 ± 0.17	0.75 ± 0.16	0.74 ± 0.18	0.252
miR-188-3p	10.34 ± 1.26	8.35 ± 1.33	6.55 ± 1.18	<0.001
miR-335-5p	0.44 ± 0.14	0.67 ± 0.16	0.88 ± 0.15	<0.001
miR-19a/b	4.04 ± 1.41	3.19 ± 1.21	2.47 ± 1.24	<0.001

### Statistical analysis

In this study, data analysis was performed using SPSS 26.0 statistical software. Categorical variables were compared using the chi-square test. For continuous variables, either one-way ANOVA or non-parametric rank-sum tests were applied, depending on whether the data followed a normal distribution. Correlation analysis was conducted using either linear correlation or Pearson correlation, as appropriate. Logistic regression analysis was employed to identify potential risk factors associated with DOP. The predictive performance of relevant factors for DOP diagnosis was assessed using receiver operating characteristic (ROC) curve analysis. *P* < 0.05 was considered statistically significant.

## Results

### Feature comparison

**Figure 1:**
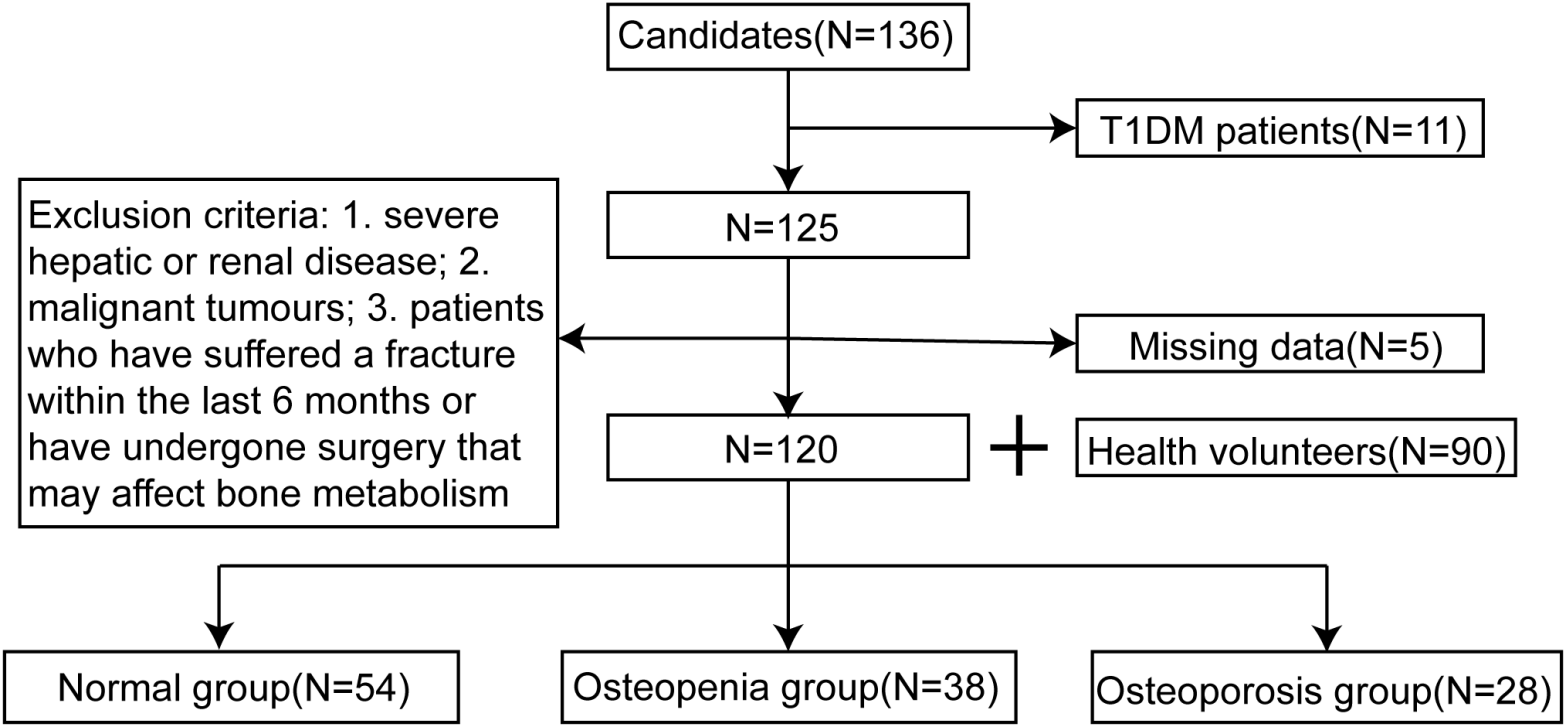
Enrollment of the study participants in the primary cohort.

**Figure 2:**
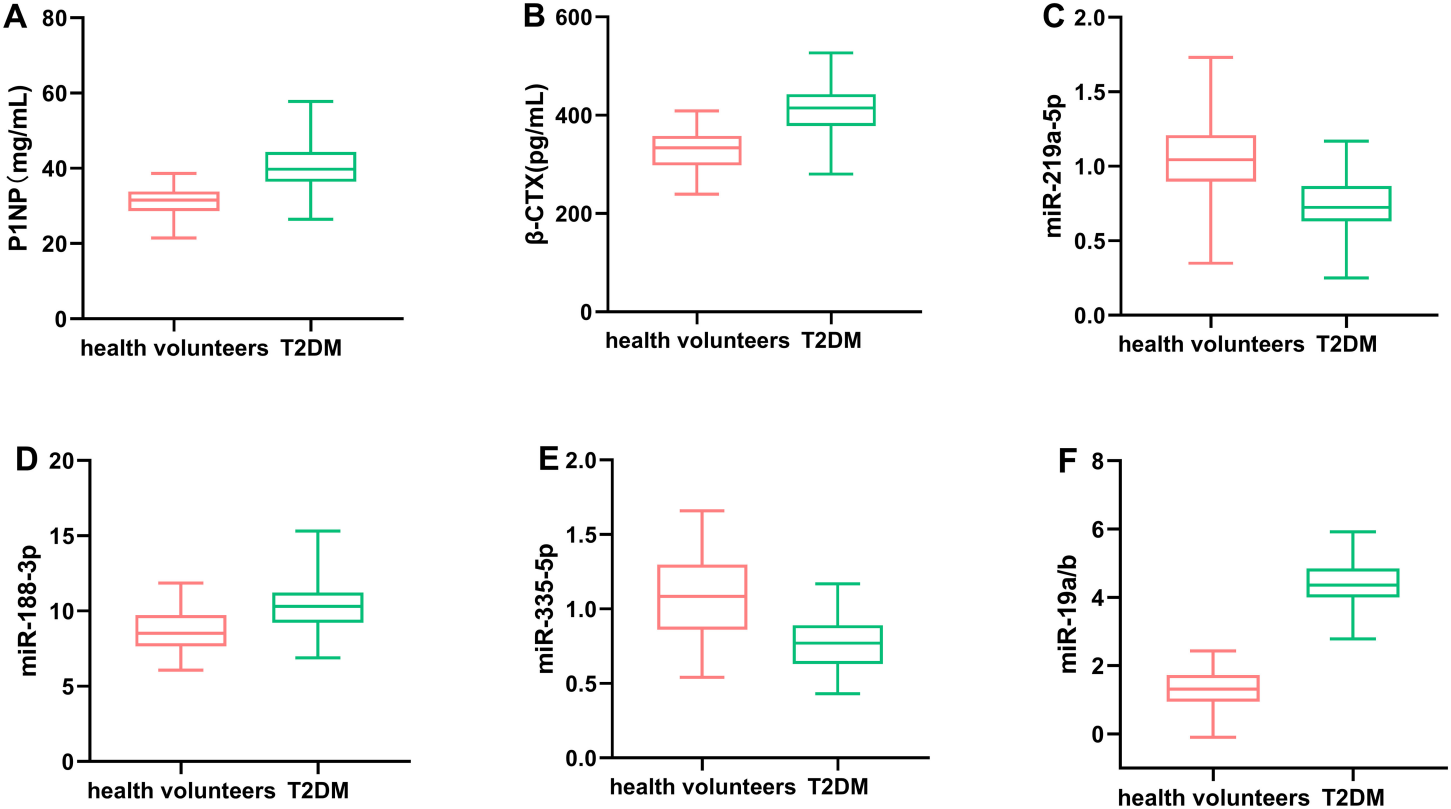
Comparison of the levels of P1NP **(A)**, β-CTX **(B)**, miR-219a-5p **(C)**, miR-188-3p **(D)**, miR-335-5p **(E)**, and miR-19a/b **(F)** in T2DM patients and healthy volunteers. Circulating levels of miR-188-3p, miR-335-5p, and miR-19a/b were significantly associated with the development of DOP in T2DM patients.

This study included 120 patients with T2DM who were treated by the department of endocrinology at our hospital during xx and 90 healthy subjects. According to the BMD values, the 120 patients were divided into three groups: normal group (54 cases), osteopenia group (38 cases), and osteoporosis group (28 cases), as shown in **Figure 1**. The differences in the levels of P1NP, β-CTX, and miRNAs in T2DM patients and healthy volunteers were analyzed, as shown in **Figure 2**. In T2DM patients, P1NP levels decreased, β-CTX levels increased, miR-219a-5p levels decreased, miR-188-3p levels increased, miR-335-5p levels decreased, and miR-19a/b levels increased.

The 120 T2MD patients were divided into three groups based on BMD. The general clinical data and laboratory tests among the three groups are compared in **Table 1**. There were significant differences in both hip and lumbar BMD among the osteoporosis group, osteopenia group, and normal group (all *P* values < 0.001). The β-CTX level also decreased with the loss of bone density, and the difference among the three groups was significant (*P* < 0.001). There was also a significant difference between each of the two groups (p<0.05). In terms of miRNA, there were statistical differences in the levels of miR-188-3p, miR-335-5p, and miR-19a/b among the three groups (all *P* values < 0.001). There was also a significant difference between each of the two groups (p<0.05). There were no significant differences in other clinical data and laboratory test indicators among the three groups.

### Correlation analysis of hip and lumbar spine mineral density with β-CTX, miR-188-3p, miR-335-5p, and miR-19a/b

In T2DM patients, hip and lumbar spine mineral density were negatively correlated with β-CTX, miR-188-3p, and miR-19a/b (*P* < 0.001), and hip and lumbar spine mineral density were positively correlated with miR-335-5p (*P* < 0.001), as shown in **Table 2**.

**Table 2:** Correlation between BMD measurement site and β-CTX, miR-188-3p, miR-335-5p, and miR-19a/b.

BMD measurement site	β-CTX		miR-188-3p		miR-335-5p		miR-19a/b	
	Correlation coefficient	*P*-value	Correlation coefficient	*P*-value	Correlation coefficient	*P*-value	correlation coefficient	*P* value
Lumbar spine	-0.529	<0.001	-0.655	<0.001	0.599	<0.001	-0.383	<0.001
Right hip	-0.433	<0.001	-0.496	<0.001	0.507	<0.001	-0.301	0.001
Left hip	-0.410	<0.001	-0.598	<0.001	0.553	<0.001	-0.433	<0.001

### Logistic regression analysis of independent predictors for osteoporosis in T2DM and ROC curve evaluation of predictive ability

**Figure 3:**
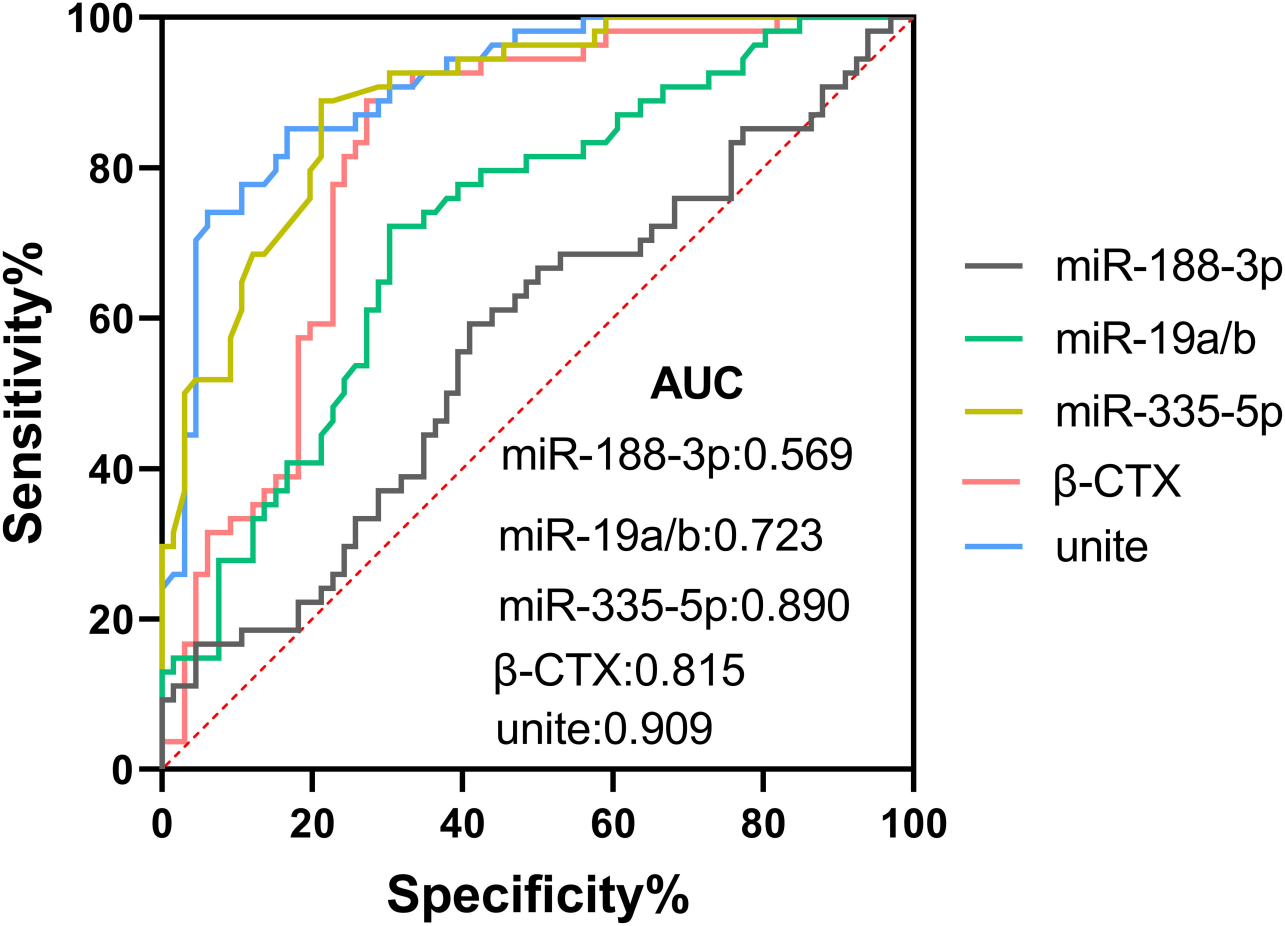
ROC curve analysis of β-CTX, miR-188-3p, miR-335-5p, and miR-19a/b as predictors of osteoporosis in T2DM, joint testing for maximum effectiveness.

Logistic regression analysis was performed to evaluate the independent predictors of DOP in T2DM patients using β-CTX and miRNAs. The model was adjusted for age, gender, BMI, duration of diabetes, and HbA1c, and variables were screened using stepwise regression. The results showed that β-CTX, miR-188-3p, miR-335-5p, and miR-19a/b were independent risk factors for DOP (OR: 1.033, 95% CIs: 1.007-1.060; OR: 2.620, 95% CIs: 1.401-4.897; OR: 0.002, 95% CIs: 0.000-0.673; OR: 2.159, 95% CIs: 1.032-4.516, respectively) (**Table 3**). The ROC curve evaluated the clinical value of these indicators in DOP, among which the combination of the four had the highest diagnostic efficacy (**Figure 3**). The AUC value of the combined diagnosis was 0.909, sensitivity (82%) and specificity (78%), and the DeLong test demonstrated that the combined index was superior to the single index (P<0.05).

**Table 3:** Logistic regression analysis reveals independent predictors of osteoporosis in patients with T2DM.

Characteristic	OR	95% CI	*P*-value
β-CTX	1.033	1.007-1.060	0.011
miR-188-3p	2.620	1.401-4.897	0.003
miR-335-5p	0.002	0.000-0.673	0.036
miR-19a/b	2.159	1.032-4.516	0.041

## Discussion​

The incidence of osteoporosis among diabetes patients ranges from 52.1% to 54.68%, with 1/3 to 2/3 of these patients showing reduced bone mineral density (BMD) (14, 15). For diabetic patients, especially elderly patients, bone fractures not only affect their quality of life, but also present treatment challenges, incur high costs, and place significant economic and social burdens on families (16–18). DOP is a preventable and treatable chronic disease with an insidious course. However, once bone loss occurs, it is often irreversible. Therefore, developing scientifically effective screening strategies for high-risk populations is crucial for the prevention, early diagnosis, and treatment of osteoporosis, as well as identifying individuals at increased risk of fractures for early intervention (19). This study identified β-CTX, miR-188-3p, miR-335-5p, and miR-19a/b as potential biomarkers for predicting the occurrence of DOP.

Dual-energy X-ray absorptiometry (DXA) is currently the gold standard for diagnosing osteoporosis. DXA can distinguish between healthy individuals, osteopenia and osteoporosis, but it cannot distinguish between different potential causes of fracture risk. The changes in bone density measured take several weeks or months to be detected (20, 21). The International Osteoporosis Foundation (IOF) recommends the use of type I procollagen N-terminal propeptide (P1NP) and serum type 1 collagen cross-linked C-terminal peptide (β-CTX) as two indicators with good sensitivity. This recommendation has also been included in the “Guidelines for the Diagnosis and Treatment of Primary Osteoporosis”. However, although bone turnover markers can reflect the status of bone metabolism to a certain extent, their specificity and sensitivity are limited by factors such as circadian rhythms and diet (22–24). The fundamental regulatory role of miRNAs in biological functions and their abnormal expression in disease pathogenesis highlight the potential of miRNAs as disease biomarkers, especially in complex syndrome conditions (15, 18, 25). In this study, we analyzed the correlation between miRNAs and BMD and found that both hip and lumbar BMD were correlated with miR-188-3p, miR-335-5p, and miR-19a/b.

Previous studies have suggested that miR-188-3p may be involved in proliferation, apoptosis, and differentiation of chondrocytes in knee osteoarthritis (26). miR-335-5p can downregulate the expression of DKK1, an inhibitor of the Wnt pathway, thereby promoting the differentiation of osteoblasts. It has also been reported that miR-335-5p is closely linked to the growth and development of osteoblasts through signaling pathways such as MAPK, Focal adhesion, and ErbB (27). miR-19a/b can improve the osteogenic differentiation of bone marrow mesenchymal stem cells in osteoporotic rats by regulating Ras homologous gene family members/Rho-associated coiled-coil protein kinase pathway proteins, promote bone mineralization, and facilitate bone formation (28). Despite initial reports of miR-19a/b and miR-335-5p in primary osteoporosis, this is the first time that the diagnostic value of their circulating levels has been validated in patients with secondary osteoporosis in T2DM and their gradient correlation with BMD has been revealed. Similar results were also observed in this study. The levels of miR-188-3p, miR-335-5p, and miR-19a/b were statistically different among the three research groups divided according to bone density loss, and ROC curve analysis showed that their combination with β-CTX can provide higher diagnostic efficacy. miRNA can provide more biological information for diagnosis and personalized treatment, and can serve as potential biomarkers for predicting osteoporosis in T2DM patients. In terms of sensitivity and specificity, although P1NP and β-CTX have been widely used as bone turnover markers in the clinic, they are affected by a variety of factors, such as circadian rhythms and diet, and there are limitations in their specificity and sensitivity. In contrast, the miRNA biomarkers identified in this study, such as miR-188-3p, miR-335-5p, and miR-19a/b, were found to be closely associated with the occurrence of DOP at the expression level in T2DM patients, and the area under the curve (AUC) values for the co-diagnosis of these miRNAs by the analysis of the working characteristics of the subjects (ROC) curve were high, and the sensitivity and specificity also showed a better level of sensitivity and specificity. The combined diagnostic index showed superior performance in predicting DOP compared with single indicators.

Moreover, the early diagnosis of DOP facilitated by the detection of these miRNAs holds significant promise in preventing fractures. By identifying individuals at high risk of DOP before the onset of clinical symptoms, clinicians can implement timely interventions, such as lifestyle modifications, pharmacological therapies, and regular bone density monitoring, to slow down or halt the progression of DOP. This proactive approach not only improves the quality of life for patients by reducing the likelihood of fractures but also alleviates the economic and social burdens associated with fracture-related complications. Therefore, the integration of miRNA biomarkers into routine clinical practice for the early diagnosis of DOP represents a crucial step forward in the management of this debilitating condition.

In contrast to previous studies, we found that the suppression of miR-335-5p expression in DOP may be affected by both the hyperglycemic microenvironment and insulin resistance, which provides a new perspective to understand the ‘dual risk factor’ of diabetic bone metabolism. It has been shown that miRNAs such as miR-188-3p, miR-335-5p and miR-19a/b are closely related to the processes of bone formation and bone resorption, and they may affect the functions of osteoblasts and osteoclasts by regulating specific signaling pathways (29). However, the specific mechanisms of the role of these miRNAs in the development of DOP in T2DM patients are not fully understood.

This study has limitations due to its retrospective design, small sample size, and lack of systematic quality control. The results may be influenced by other factors, leading to variability in conclusions. In addition, the small sample size of the osteoporosis subgroup in this study may have affected the validity of analyses of rare covariates (e.g., severe vitamin D deficiency). Unfortunately, due to the limitations of the study design and scope of data collection, the effect of menopausal status on miRNA expression and bone metabolic markers was not systematically analyzed in this paper. Postmenopausal women are known to be at high risk for osteoporosis and fractures. Hormonal changes associated with menopause can have a significant impact on bone metabolism, which may alter the expression patterns of miRNAs and other DOP-related biomarkers. Therefore, the results of this study, while valuable, may not fully reflect the complexity of DOP in postmenopausal women with T2DM. Future studies with a specific focus on postmenopausal women will allow us to better understand the specific role of miRNAs in DOP in this high-risk subgroup and develop more targeted screening and intervention strategies. Future research will aim to validate these findings in multi-center, large-sample, prospective studies. Although this study controlled for confounders as much as possible through baseline characteristic comparisons and covariate adjustment, unrecorded variables (e.g., exercise intensity, dietary calcium intake) may have influenced the results. Further validation in conjunction with a prospective design is needed in the future. Additionally, U6 may not be suitable as a universal internal reference across all pathological conditions. Future studies will incorporate the combined use of multiple internal reference genes, such as miR-16 and RNU48, to enhance methodological rigor and standardization.

## Conclusion

The findings of this study demonstrate that circulating levels of miR-188-3p, miR-335-5p, and miR-19a/b are significantly associated with the development of DOP in patients with T2DM. These miRNAs exhibit promising potential as biomarkers for early diagnosis of DOP.

## Data Availability

The original contributions presented in the study are included in the article/supplementary material. Further inquiries can be directed to the corresponding author.
